# Assessment of quality of life determinants in hemodialysis patients of a developing country: A cross-sectional study during ongoing COVID-19 pandemic

**DOI:** 10.1097/MD.0000000000029305

**Published:** 2022-08-05

**Authors:** Muhammad Sohaib Asghar, Muhammad Nadeem Ahsan, Pooran Mal, Muhammad Junaid Tahir, Farah Yasmin, Khabab Abbasher Hussien Mohamed Ahmed

**Affiliations:** a Department of Internal Medicine, Dow University of Health Sciences–Ojha Campus, Karachi, Pakistan; b Department of Nephrology, Dow University of Health Sciences–Ojha Campus, Karachi, Pakistan; c Department of Nephrology, Liaquat University of Medical & Health Sciences, Karachi, Pakistan; d Department of Medicine, Lahore General Hospital, Lahore, Pakistan; e Department of Internal Medicine, Dow Medical College, Dow University of Health Sciences, Karachi, Pakistan; f University of Khartoum, Faculty of Medicine, Khartoum, Sudan.

**Keywords:** CKD, coronavirus, dialysis, ESRD, outbreak, quality of life

## Abstract

**Background and Objectives:**

Patients of end-stage renal disease are prone to have a very low quality of life (QoL). Variety of factors influence the QoL among sufferers of chronic kidney disease comprising of type of dialysis, sufficiency/adequacy of dialysis, and associated burden of disease. We conducted this study amidst the pandemic to determine the associated factors for poor QoL in hemodialysis patients during the ongoing pandemic.

**Patients and Methods:**

This cross-sectional study was conducted in a hemodialysis unit of a tertiary care hospital. A total of 118 participants responded to the validated questionnaire of Quality of Life Index-dialysis version-III (QLI). Higher scores signify good QoL, total scores are further categorized into subgroups desirable, relatively desirable and undesirable.

**Results:**

The mean age of the participants was 57.36 ± 10.03 years and mean body mass index of 26.73 ± 5.54 kg/m^2^. The mean total QoL of the study population was found quite low (12.99 ± 5.89). Majority of respondents fell in undesirable category of QoL (49.2%). Total QoL (*P* = 0.004) and subscale health/functioning (*P* = 0.003) were significantly lower in females. All the subscales along with total QoL scores were found lower in twice-weekly dialyzed patients (*P* < 0.001). Marital status (*P* = 0.049) and twice-weekly dialysis (*P* < 0.001) were found significant with undesirable QoL. On multivariate analysis, significant determinants of undesirable QoL were twice-weekly dialysis (*P* = 0.001), catheter access (*P* = 0.034), phosphate (*P* = 0.005) and uric acid (*P* = 0.006).

**Conclusion:**

Inadequate dialysis due to lesser frequency per week leading to poorly cleared toxic substances were most significant contributors of poor QoL in our study.

**What Was Known/What This Study Adds**

**What Was Known**

- Patients of end-stage renal disease (ESRD) are prone to have a very low quality of life (QoL).
- Variety of factors influence the QoL among sufferers of CKD.
- Hemodialysis is also recognized with affecting the quality of life and sleep in patients along with the chronic kidney disease itself.

**What This Study Adds**

- Lesser frequency of dialysis/inadequate dialysis was found the most significant contributor towards poor QoL.
- Certain biochemical markers were the major contributor towards poor QoL like uric acid, serum phosphate and others.
- Obtaining constant COVID-19 updates and fear of contracting the virus were additional associated factors on multivariate analysis.

## 1. Introduction

Hemodialysis is preferably opted modality of treatment for survival by more than 70% of patients with end-stage renal disease (ESRD).^[1,2]^ Chronic kidney disease (CKD) imparts a pronounced negative effect on quality of life (QoL) of sufferers prominently due to association with impairment and restrictions in all aspects of daily life.^[1,3]^ Patients with ESRD are prone to have very low QoL.^[4]^ Variety of factors influence the QoL among sufferers of CKD undergoing hemodialysis comprising of type of dialysis (hemodialysis and peritoneal dialysis), sufficiency of dialysis, daily dialysis, night shift of dialysis, depression and anxiety associated with burden of disease, frequent hospital admissions, three times a week visits to dialysis centers, decline in physical fitness and increasing age.^[5,6]^

QoL in patients of ESRD undergoing dialysis is also affected by multiple clinical manifestations (i.e., vomiting, nausea, decreased appetite, exhaustion, muscle cramps, chronic pain, and sleep disturbances).^[1,2]^ Pakistan has a predicted number of 150 patients with CKD per annum per million, with estimated value of 16,000 patients each year.^[5]^ Sufferers of ESRD initially are scheduled thrice weekly irrespective of residual renal status.^[7]^ The frequent sessions of dialysis exert prominent effect on QoL.^[8]^ Maximum population of United States of America (USA), Europe, and Japan undergo a traditional thrice weekly course of dialysis while patients of Thailand undergo twice-weekly course of dialysis due to economic restrictions, curtail the decline of QoL.^[8]^ A thrice-weekly plan of dialysis ensures the prolong duration of treatment and higher Kt/V were associated with decline in rate of mortalities due to adequate dialysis.^[8]^

Hemodialysis patients from Taiwan showed lower scores of QoL when compared with patients undergoing peritoneal dialysis.^[9]^ The reported quality of life using the same scale in our study was reported to be satisfactory in the Michigan population on peritoneal dialysis.^[10]^ Hence, QoL is significantly more of a concern in hemodialysis patients. A study demonstrates empowerment counseling sessions of hemodialysis patients improve self-efficacy, quality of life, and clinical status.^[11]^ On the contrary, currently, newer therapies are employed amidst the pandemic and compared with hemodialysis, QoL generally appears to be superior with convective therapies.^[12–14]^ In 1 randomized controlled trial conducted in the Netherlands, QoL improved on hemofiltration versus low-flux hemodialysis.^[12]^ In a meta-analysis, QoL significantly improved using an unvalidated scale in patients on hemodiafiltration therapy compared with those on hemodialysis therapy.^[15]^

Hence, we conducted this study to determine the associated factors for poor QoL in hemodialysis patients during the coronavirus disease 2019 (COVID-19) ongoing pandemic. The secondary aim of this study is to associate biochemical markers with QoL among sufferers of ESRD undergoing either twice weekly or thrice weekly sessions of dialysis.

## 2. Material and Methods

### 2.1. Study design and settings

This observational study was conducted during the ongoing COVID-19 pandemic as cross-sectional analysis in a hemodialysis unit of a tertiary care hospital during the months of May till October 2021.

*Participants and variables:* More than 200 patients are registered for hemodialysis in the unit. After taking ethical approval from the institutional review board, assessment of QoL by using the Ferrans and Powers Quality of Life Index-dialysis version-III (QLI) was undertaken with consent of the participants.^[16–18]^ The second part of the questionnaire was recording patient factors and biochemical data of the patients including age, body mass index (BMI), duration of hemodialysis onset, frequency of dialysis per week, marital status, comorbidities, line access [Arteriovenous (AV) fistula or catheter], and current laboratory values of hemoglobin, albumin, uric acid, serum phosphate, serum calcium, and parathyroid hormone (PTH) levels. The most recent laboratory markers were considered for preceding 1 month prior to inclusion in the study.

### 2.2. Data sources/instrument and measurement

The different versions of QLI were available online and showed precision to great degrees in previously conducted studies with a Cronbach alpha coefficient of 0.93.^[19,20]^ The QLI is divided into 2 parts with 35 items each; first part being satisfaction using a 6-point Likert scale (1 = very dissatisfied, 6 = very satisfied), and the second part is the importance (1 = very unimportant, 6 = very important). The items are grouped into 4 subscales: health/functioning, socioeconomic, psychological/spiritual, and family.^[21]^ Higher scores signify good QoL of the patient and vice versa.^[17]^ The range for the final scores is same for all the subscales and total score, which is 0 to 30. Total scores are further categorized into 3 subgroups: desirable (score: 20–30), relatively desirable (score: 10–19) and undesirable (score: 0–9).^[18]^

### 2.3. Bias

Some questions also depend on patients’ perception regarding certain beliefs; hence they might affect the results.

### 2.4. Study size

A sample size of 132 was calculated via OpenEpi sample size calculator,^[22]^ in which we used 5% as a margin of error, 95% as confidence interval (CI), 200 as population size (currently enrolled number of patients in hemodialysis unit) and anticipated frequency of outcome factor in the population of 50%. Hence, the questionnaire was made available in printed form with translation in local language alongside for better understanding. At least, 180 patients were approached for filling out the responses, out of which 137 agreed to participate giving a response rate of 76.1%. A total of 19 questionnaires were excluded due to incomplete responses. Hence, a total of 118 respondents (participants) were recruited in the analysis.

### 2.5. Statistical methods

Subsequently, data were tabulated, coded then analyzed using the computer program for Windows IBM SPSS (Statistical Package for Social Sciences) version 25.0, Armonk, NY: IBM Corporation.

### 2.6. Quantitative variables

Descriptive statistics were calculated in the form of mean ± standard deviation, 95% CI, range, median and interquartile range (IQR) and frequency (percentage). In the statistical comparison between the different groups, the significance of difference was tested using either Mann-Whitney used to compare between different groups of nonparametric data and inter-group comparison of categorical data was performed by using Pearson chi-square test or Fisher exact test as indicated. Multivariate linear regression was conducted for only the continuous descriptive variables reported unstandardized and standardized coefficients, while multivariate regression analysis was conducted for all the qualitative variables. Crude odds ratio (OR) and adjusted odds ratio (AOR) were reported subsequently. A *P* value <0.05 was considered statistically significant.

## 3. Results

### 3.1. Sociodemographic characteristics of participants

A total of 118 responses were including in the analysis having a mean age of 57.36 ± 10.03 years and mean BMI of 26.73 ± 5.54 kg/m^2^. About 48.3% of participants were enrolled for twice-weekly dialysis and rest 51.7% for thrice weekly, majority dialyzed via AV fistula access (79.7%). Around 55.1% comprised of men, 66.9% were married, 74.6% hypertensive, 56.8% diabetic, and 11.9% suffered from ischemic heart disease (IHD). The mean values of all laboratory markers are shown in Table 1.

**Table 1:** Baseline data of the study population (n = 118).

Characteristics	Frequency/descriptive
Mean age (yr)	57.36 ± 10.03
Mean body mass index (kg/m^2^)	26.73 ± 5.54
Mean duration of hemodialysis onset (yr)	3.87 ± 3.45
>2 years of hemodialysis onset	63 (53.4%)
<2 years of hemodialysis onset	55 (46.6%)
Male gender	65 (55.1%)
Female gender	53 (44.9%)
Frequency of dialysis: twice weekly	57 (48.3%)
Frequency of dialysis: thrice weekly	61 (51.7%)
Marital status: single	39 (33.1%)
Marital status: married	79 (66.9%)
Diabetes	67 (56.8%)
Hypertension	88 (74.6%)
Ischemic heart disease	14 (11.9%)
Line access: femoral/subclavian catheter	24 (20.3%)
Line access: arteriovenous fistula	94 (79.7%)
Mean hemoglobin (g/dL)	9.69 ± 1.11
Mean albumin (g/dL)	3.53 ± 0.52
Mean uric acid (mg/dL)	6.58 ± 1.03
Mean phosphate (mg/dL)	5.59 ± 2.28
Mean calcium (mg/dL)	8.26 ± 0.91
Mean parathyroid hormone (pg/mL)	557.30 ± 453.50

### 3.2. Descriptive data

**Figure 1 & 2.**
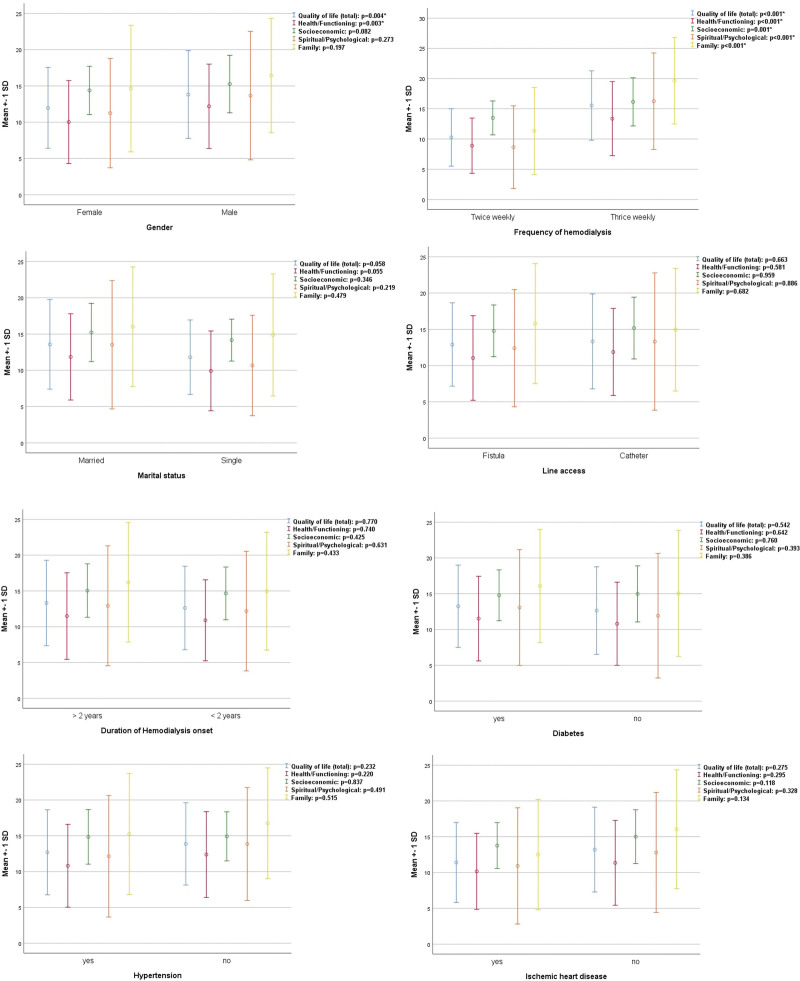
Inferential statistics involving nonparametric distribution of quality of life scores among study variables.

**Figure 3 & 4.**
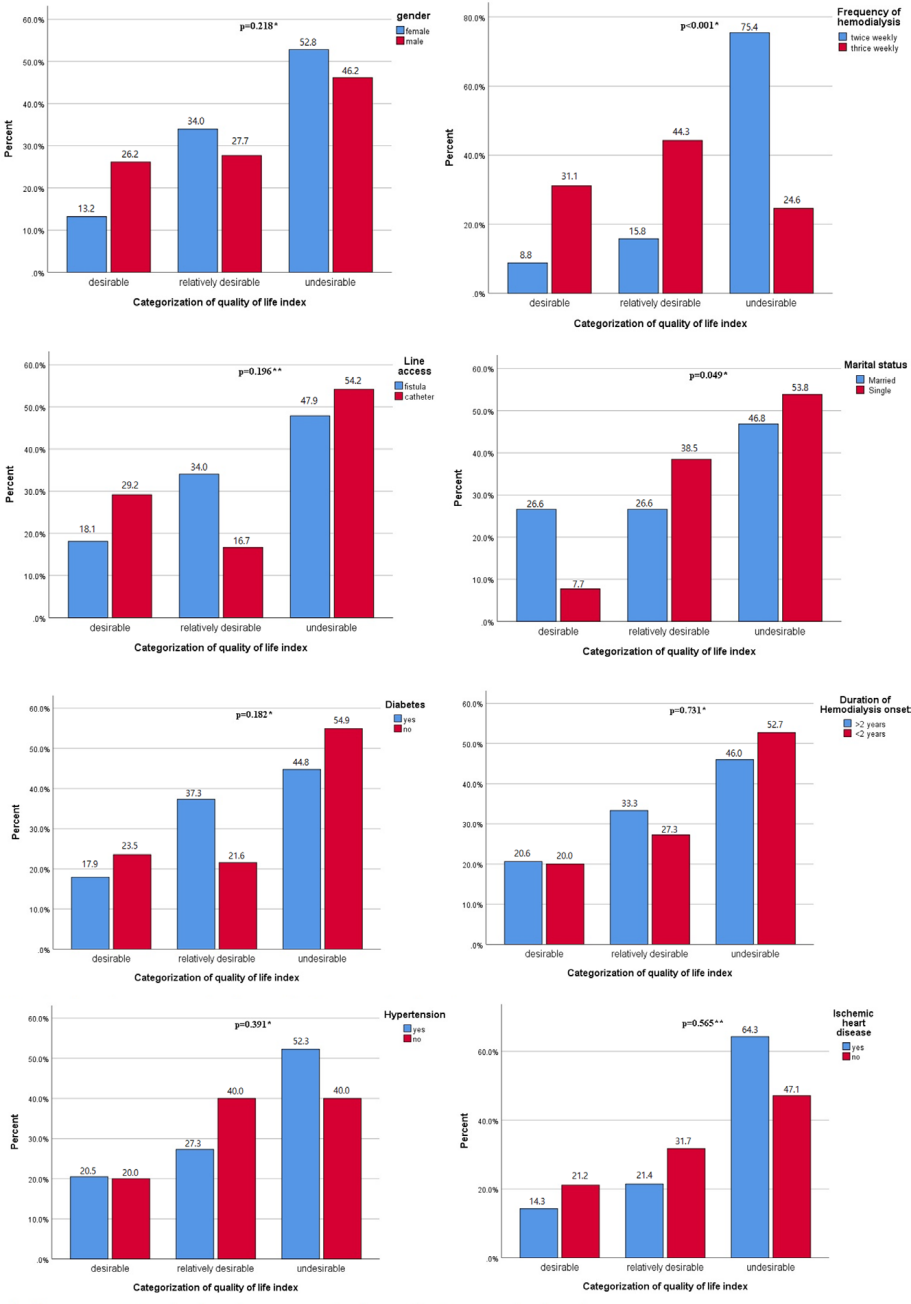
Categorical distribution of quality of life determinants into subcategories of desirable, relatively desirable and undesirable.

The mean total QoL of the study population was found quite low (12.99 ± 5.89), with subscales scoring of 11.21 ± 5.85 (Health/Functioning), 14.86 ± 3.70 (Socioeconomic), 12.58 ± 8.34 (Spiritual/Psychological), and 15.63 ± 8.28 (Family). Majority of respondents fell in undesirable category of QoL (49.2%) followed by relatively desirable (30.5%) and desirable (20.3%), as shown in Table 2. In inferential statistics, total QoL (*P* = 0.004) and subscale health/functioning (*P* = 0.003) were significantly lower in females. All the subscales along with total QoL scores were found lower in twice-weekly dialysis patients (*P* < 0.001), as shown in Figures 1 and 2. With respect to diabetes, hypertension and IHD, no such differences were observed. Among categorization, marital status (*P* = 0.049) and frequency of dialysis (*P* < 0.001) were found significant, as shown in Figures 3 and 4. Among them, 53.8% unmarried and 75.4% twice weekly dialyzed patients had undesirable QoL. Among others, line access via catheter (54.2%), duration of dialysis (52.7%) and females (52.8%) were having undesirable QoL.

**Table 2:** Quality of life index scores of the study population (n = 118).

Scoring/categorization	Subscales	Mean ± SD	Median	IQR	Range	95% CI
Quality of life index scores	Health/functioning	11.21 ± 5.85	7.82	12.28	17.22	10.14–12.28
	Socioeconomic	14.86 ± 3.70	12.81	4.00	11.00	14.19–15.54
	Spiritual/psychological	12.58 ± 8.34	10.64	14.00	22.14	11.05–14.10
	Family	15.63 ± 8.28	16.80	19.40	22.50	14.12–17.14
	Quality of life total	12.99 ± 5.89	11.68	11.01	15.81	11.91–14.06
Quality of life (total) categorization	Categories	Frequency		Percentage		
	Desirable (score: 20–30)	n = 24		20.3%		
	Relatively desirable (score: 10–19)	n = 36		30.5%		
	Undesirable (score: 0–9)	n = 58		49.2%		

## 4. Outcome data and main results

All the descriptive variables were analyzed for multiple linear regression with QoL score (total) as dependent variable. Increased age was inversely related to QoL score (*P* = 0.031), while uric acid levels (*P* < 0.001), serum phosphate (*P* = 0.044) and BMI (*P* < 0.001) were directly associated with QoL score as shown in Table 3. For all categorical variables, univariate and multivariate regression was conducted with desirable QoL as reference category. On univariate model, BMI with OR: 4.857 (1.584–14.890), marital status with OR: 0.200 (0.050–0.794), lower hemoglobin with OR: 0.280 (0.085–0.925), high phosphate with OR 3.788 (1.275–11.254) and calcium with OR: 3.720 (1.060–13.050) were associated with relatively desirable QoL. When adjusted for all the factors, hemoglobin with AOR 0.109 (0.013–0.945), marital status with AOR: 0.109 (0.013–0.945), phosphate with AOR 18.207 (1.880–76.291) and calcium with AOR: 28.468 (1.825–44.104) remained associated with relatively desirable QoL as shown in Table 4.

**Table 3:** Multiple linear regression of all descriptive variables with dependent variable of quality of life index (total).

Model	Unstandardized coefficients (B)	Standard error	Standardized coefficients (β)	*t*-statistic	*P* value	95% Confidence Interval for B
(Constant)	35.591	9.224	–	3.859	<0.001*	17.309–53.874
Age	–0.148	0.068	–0.251	–2.184	0.031*	–0.282 to –0.014
Hemoglobin	–1.044	0.531	–0.198	–1.967	0.052	–2.096 to 0.008
Albumin	0.795	1.048	0.070	0.758	0.450	–1.283 to 2.872
Uric acid	–1.940	0.512	–0.341	–3.793	<0.001*	–2.955 to –0.926
Phosphate	–0.486	0.238	–0.188	–2.040	0.044*	–0.959 to –0.014
Calcium	–0.410	0.667	–0.064	–0.614	0.540	–1.732 to 0.912
Parathyroid hormone	–0.002	0.001	–0.117	–1.193	0.236	–0.004 to 0.001
Duration of hemodialysis onset	–0.087	0.156	–0.051	–0.557	0.579	–0.397 to 0.223
BMI	0.495	0.128	0.466	3.860	<0.001*	0.241 to 0.749

**Table 4:** Univariate and multivariate logistic regression model for quality of life in hemodialysis patient (n = 118).

Variables	Relatively desirable (score: 10-19)		Undesirable (score: 0-9)	
	Crude OR (95%CI)	AOR (95%CI)	Crude OR (95%CI)	AOR (95%CI)
Gender				
Male	1.000	1.000	1.000	1.000
Female	2.429 (0.812–7.268)	0.474 (0.060–3.725)	2.267 (0.818–6.285)	1.065 (0.147–7.696)
*P* value	0.113	0.478	0.116	0.950
Age				
<50 years	1.000	1.000	1.000	1.000
>50 years	1.800 (0.588–5.511)	0.807 (0.112–5.834)	1.575 (0.575–4.312)	0.650 (0.106–4.002)
*P* value	0.303	0.832	0.377	0.643
Frequency of hemodialysis				
Thrice weekly	1.000	1.000	1.000	1.000
Twice weekly	1.069 (0.345–3.309)	3.901 (0.639–23.813)	4.985 (1.767–14.062)	24.088 (3.974–46.011)
*P* value	0.908	0.140	0.002*	0.001*
BMI				
<25.0 kg/m^2^	1.000	1.000	1.000	1.000
>25.0 kg/m^2^	4.857 (1.584–14.890)	0.385 (0.029–5.184)	3.206 (1.153–8.909)	0.798 (0.073–8.679)
*P* value	0.006*	0.472	0.026*	0.853
Line access				
Fistula	1.000	1.000	1.000	1.000
Catheter	1.167 (0.347–3.924)	0.445 (0.038–5.235)	1.815 (0.566–5.822)	14.164 (1.227–63.439)
*P* value	0.803	0.520	0.316	0.034*
Marital status				
Single	1.000	1.000	1.000	1.000
Married	0.200 (0.050–0.794)	0.109 (0.013–0.945)	0.252 (0.067–0.945)	0.187 (0.024–1.482)
*P* value	0.022*	0.044*	0.041*	0.112
Duration of dialysis onset				
<2 years	1.000	1.000	1.000	1.000
>2 years	1.185 (0.418–3.355)	0.870 (0.155–4.875)	0.846 (0.326–2.196)	0.629 (0.117–3.377)
*P* value	0.750	0.874	0.731	0.589
Diabetes				
Absent	1.000	1.000	1.000	1.000
Present	1.185 (0.418–3.355)	2.969 (0.555–15.890)	1.199 (0.460–3.124)	4.220 (0.785–22.695)
*P* value	0.750	0.204	0.711	0.093
Hypertension				
Absent	1.000	1.000	1.000	1.000
Present	0.824 (0.269–2.525)	0.146 (0.020–1.077)	1.759 (0.587–5.275)	0.338 (0.047–2.443)
*P* value	0.734	0.059	0.313	0.282
Ischemic heart disease				
Absent	1.000	1.000	1.000	1.000
Present	1.000 (0.154–6.480)	0.158 (0.008–2.967)	2.020 (0.403–10.134)	4.194 (0.331–53.122)
*P* value	1.000	0.217	0.393	0.268
Hemoglobin				
>9 g/dL	1.000	1.000	1.000	1.000
<9 g/dL	0.280 (0.085–0.925)	0.045 (0.004–0.457)	0.580 (0.216–1.561)	0.269 (0.036–2.032)
*P* value	0.037*	0.009*	0.281	0.203
Albumin				
>3.4 g/dL	1.000	1.000	1.000	1.000
<3.4 g/dL	1.400 (0.495–3.956)	0.331 (0.046–2.402)	0.871 (0.336–2.257)	0.229 (0.031–1.706)
*P*-value	0.526	0.274	0.776	0.150
Uric acid				
<6.5 mg/dL	1.000	1.000	1.000	1.000
>6.5 mg/dL	1.667 (0.581–4.779)	4.420 (0.828–23.591)	2.536 (0.952–6.755)	10.723 (2.001–57.467)
*P* value	0.342	0.082	0.063	0.006*
Phosphate				
<4.5 mg/dL	1.000	1.000	1.000	1.000
>4.5 mg/dL	3.788 (1.275–11.254)	18.207 (1.880–76.291)	4.020 (1.477–10.941)	22.043 (2.578–88.464)
*P* value	0.017*	0.012*	0.006*	0.005*
Calcium				
>8.6 mg/dL	1.000	1.000	1.000	1.000
<8.6 mg/dL	3.720 (1.060–13.050)	28.468 (1.825–44.104)	1.720 (0.624–4.742)	5.567 (0.441–70.341)
*P* value	0.040*	0.017*	0.295	0.185
Parathyroid hormone				
<300 pg/mL	1.000	1.000	1.000	1.000
>300 pg/mL	2.071 (0.634–6.767)	0.201 (0.024–1.702)	1.731 (0.606–4.943)	0.136 (0.017–1.099)
*P* value	0.228	0.141	0.306	0.061
Obtaining constant COVID-19 updates				
Yes	1.000	1.000	1.000	1.000
No	0.169 (0.102–0.281)	0.439 (0.218–0.886)	5.921 (3.559–9.851)	2.277 (1.129–4.592)
*P* value	<0.001*	0.001*	0.005*	0.001*
Fear of contracting COVID-19				
Yes	0.678 (0.410–1.120)	0.446 (0.215–0.927)	1.475 (0.893–2.438)	2.240 (1.079–4.648)
No	1.000	1.000	1.000	1.000
*P* value	0.069	0.031*	0.051	0.042*

Significant determinants of undesirable QoL were twice the weekly frequency of dialysis with OR: 4.985 (1.767–14.062), BMI >26 kg/m^2^ with OR: 3.206 (1.153–8.909), marital status with OR: 0.252 (0.067–0.945), and high serum phosphate with OR: 4.020 (1.477–10.941) on univariate analysis. While after adjusted for all factors in multivariate analysis, twice-weekly frequency of dialysis with AOR 24.088 (3.974–46.011), line access via catheter with AOR: 14.164 (1.227–63.439), elevated phosphate with AOR 22.043 (2.578–88.464) and uric acid levels with AOR: 10.723 (2.001–57.467) were found associated with undesirable QoL. Obtaining constant COVID-19 updates and fear of contracting the virus were additional associated factors on multivariate analysis for relatively desirable and poor QoL as shown in Table 4.

## 5. Discussion

Many factors related to QoL of hemodialysis patients were found in the literature. Starting with perceived social support, Iranian population showed direct correlation of this factor with the same index of QoL used in our study in all the subgroups.^[23]^ Poor nocturnal quality sleep and increased daytime sleepiness are associated with decreased quality of life in hemodialysis patients of USA.^[24]^ Data from United Arab Emirates (UAE) suggested lower educational level and presence of chronic illness had the strongest impact on poor QoL.^[25]^ Hemodialysis patients from Saudi Arabia showed higher QoL scores in those undergoing afternoon shift dialysis; furthermore, male gender, employed patients, and nondiabetics also showed higher scores.^[26]^ Data from Bahrain suggested educational level, urban residence, and marital status determine poor QoL.^[27]^ Only age and educational level had a negative impact on quality of life of Iranian population on hemodialysis with either hepatitis B, or C seropositivity.^[28]^ A systematic review from Iran also suggested similar findings when compared with normal population and other chronic illnesses like diabetes and cardiac disease.^[29]^

Since frequency of dialysis was the major factor contributing to poor QoL in our patients, most of the comparisons drawn from the other studies revolve around this factor. The frequency of hemodialysis sessions has a vital role in the survival and prevalence of complications in patients enduring ESRD.^[30]^ Abundant studies conducted regarding frequency of twice or thrice sessions a week in sufferers of CKD reported age range of 50–60 years in maximum individuals.^[8,30–33]^ No gender discrimination was observed in encountering CKD among both genders, while in terms of sessions of dialysis, increased frequency of twice-weekly dialysis was detected among females.^[8,30–33]^Significant frequency of thrice-weekly dialysis was reported in males.^[8]^ Primary causes of CKD were diabetes mellitus and glomerulonephritis in multiple studies.^[7,30,32,33]^ Significant prevalence of cardiovascular diseases, stroke, dementia, hemiplegia, chronic pulmonary diseases were detected in patients of CKD undergoing 3 sessions of dialysis per week in numerous studies,^[30–32]^while no difference regarding comorbidities was reported by a trivial study.^[33]^ Higher glomerular filtration rate (GFR), urine output, and decreased creatinine levels were observed in sufferers undergoing thrice-weekly dialysis in study regulated by Park et al^[7]^ On contrary, study regulated within population of USA reported lower serum creatinine levels in individuals opting for twice-weekly sessions of dialysis when compared with thrice-weekly sessions.^[32]^ Thaweethamcharoen et al, reported no difference in creatinine levels.^[8]^ Hanson et al, reported decreased creatinine levels and elevated levels of albumin as distinctive features encountered in individuals undergoing twice-weekly sessions of dialysis.^[32]^

According to the Chinese renal data system, inhabitants of China suffering from ESRD undergo twice-weekly sessions of dialysis, factors leading to this management plan are patient comorbidities, residual function status, desire of gradual initiation of dialysis and insurance status.^[31]^ Previous studies regulated in a similar pattern concluded that twice-weekly sessions of dialysis were utilized for patients of old age, females, sufferers with low BMI and patients with residual renal function at initiation of renal replacement therapy on contrast this regimen is not recommended recently by international guidelines or in sufferers with urea clearance of <2 ml/min.^[32,34]^ Multiple studies documented the comparison of clinical outcomes between fewer weekly sessions of dialysis versus thrice-weekly plan of dialysis concluding that fewer consecutive sessions of dialysis favor the conservation of residual renal function and do not result in decline of QoL and patient survival.^[7]^ Lower clearance rate, increased Kt/V, elevated blood urea nitrogen levels (BUN), PTH and phosphate levels while decreased levels of calcium were detected in patients settling for twice-weekly program of dialysis in a study conducted within inhabitants of China and Thailand.^[8,31]^ Anemia was prominently suffered by patients with increased frequency of thrice-weekly sessions of dialysis,^[30]^ while no statistically significant difference was observed in another study conducted in similar fashion.^[8]^ Twice-weekly sessions of dialysis were popular among older Caucasian women and individuals suffering from Human Immunodeficiency Virus (HIV) and Acquired Immunodeficiency Syndrome (AIDS) according to study mentioned above.^[32]^ Majority of studies stated better QoL among individuals adjusting with twice-weekly sessions of dialysis as compared to thrice-weekly sessions,^[2,7,30,33]^ while a study recorded no difference of QoL among both frequencies of dialysis.^[8,31]^Impaired mental status, disturbances of sleep, decline in sexual and cognitive function was prominent among patients with thrice-weekly schedule of dialysis.^[7]^ Social interaction and support were prevalent within individuals with twice-weekly sessions of dialysis.^[2,7]^ Decline in mortality rate was observed in patients with twice-weekly sessions of dialysis,^[32]^ while prevalence of infection was high among patients with thrice-weekly sessions of dialysis.^[33]^ No difference within BMI, nutritional status and weight in association with frequency of dialysis was detected in patients of CKD.^[7,33]^

Other significant factors contributing to improved QoL in hemodialysis patients were illness acceptance and enough disease knowledge.^[1,35]^ Data from Ethiopia suggested socioeconomic and educational status on top of higher hemoglobin levels were significant determinants for improved QoL.^[2]^ Higher BMI and duration of dialysis onset >5 years were associated with poor QoL in an Iranian population; however, these variables are insignificant in our findings.^[35]^ Previous studies from Pakistan have suggested presence of diabetes and duration of dialysis onset negatively correlating with QoL.^[5]^ But no association was found in frequency of dialysis per week with QoL in their study participants contrary to our findings. Data from Poland suggested female gender, elderly age and fatigue during dialysis were profound determinants for poor QoL,^[6]^ as opposed to socioeconomic and educational well-being for good QoL.^[3,6]^A randomized trial from China rendered twice-weekly dialysis more favorable toward quality of life with certain socioeconomic and social support factors related to it, thus opposing our results,^[30]^ while a study from India suggested equal effects of twice and thrice-weekly dialysis on QoL.^[33]^ In another study, less frequent dialysis sessions per week have a higher score of QoL on physical and mental scales than more frequent sessions, again contradicting our results.^[2]^ More recently, COVID-19 pandemic has also affected the QoL of hemodialysis patients as evident by our results; however, not much literature is available on it to compare our results.

## 6. Limitations

There were some limitations of the current study. The study sample was not that adequate to represent a larger hemodialysis population, but a convenience sample from a single institution. The study was cross-sectional hence not signifying the causal relationship between the factor variables and QoL. Finally, because the data was collected during the dialysis unit visits accompanying by their family members, influence of those family members in answering the questions of the participants could not be certainly ruled out. Some questions also depend on patients’ perception regarding certain beliefs; hence they might affect the results. The strengths of the study were validated tools utilized, and inclusion of biochemical parameters in the analysis which are usually not a part of such surveys while reporting QoL in hemodialysis patients.

## 7. Conclusions

Our study concluded many determinants associated with relatively desirable and undesirable QoL using a validated index. Certain biochemical markers were the major contributor towards poor QoL, which can be asserted to the fact these molecules are poorly cleared from the bloodstreams by various factors including poor compliance to dialysis or lesser Kt/V. Hence, lesser frequency of dialysis implying to inadequate dialysis was found the most significant contributor towards poor QoL in our study population.

## Acknowledgment

An expert statistician from Dow University Hospital has reviewed the results.

## Footnotes

* Indicates significant *P* value of <0.05.

* Indicates *P* value significant at <0.05.
